# Can Tirofiban Improve the Outcome of Patients With Acute Ischemic Stroke: A Propensity Score Matching Analysis

**DOI:** 10.3389/fneur.2021.688019

**Published:** 2021-09-13

**Authors:** Lingxin Cai, Xiaobo Yu, Jun Yu, Jing Xu, Liang Xu, Chenhan Ling, Min Lou, Cheng Yu, Cong Qian

**Affiliations:** ^1^Department of Neurological Surgery, The Second Affiliated Hospital of Zhejiang University School of Medicine, Hangzhou, China; ^2^Department of Neurology, The Second Affiliated Hospital of Zhejiang University School of Medicine, Hangzhou, China; ^3^Department of Neurosurgery, The Second People's Hospital, Quzhou, China

**Keywords:** tirofiban, acute ischemic stroke, propensity score matching, mechanical thrombectomy, posterior circulation stroke

## Abstract

**Objective:** To evaluate the efficacy and safety of tirofiban for patients with acute ischemic stroke (AIS), especially posterior circulation stroke (PCS).

**Methods:** We enrolled consecutive patients with AIS who suffered large artery occlusion (LAO) and underwent mechanical thrombectomy (MT) between January 2016 and May 2020. Patients were divided into two groups according to whether tirofiban was used during MT. The primary efficacy outcome was a favorable functional outcome, defined as a modified Rankin Scale (mRS) score of 0–2 at 3 months. The safety outcomes were the rate of mortality at 3 months and the presence of intracranial hemorrhage (ICH) and symptomatic intracranial hemorrhage (sICH). Cohorts were balanced using 1:1 propensity score matching (PSM). Subgroup analysis was further performed to compare the efficacy and safety of tirofiban between the anterior circulation stroke (ACS) and PCS groups.

**Results:** A total of 292 patients were eligible for this study and divided into the tirofiban group (*n* = 51) and the no-tirofiban group (*n* = 241). In the propensity-score-matched cohort, the tirofiban group had a higher rate of favorable outcomes than the no-tirofiban group (49.0 vs. 25.5%, *p* = 0.014), and the mortality at 3 months showed a greater downward trend in the tirofiban group than the no-tirofiban group (15.6 vs. 33.3% *p* = 0.064). The risk of sICH and ICH was the same between the tirofiban and control groups (17.6 vs. 27.4% *p* = 0.236, 31.3 vs. 45.1% *p* = 0.154, respectively). Tirofiban use was predictive of favorable outcomes [adjusted odds ratio (aOR) = 2.87, 95% confidence interval (CI) 1.52–6.44, *p* = 0.043] after multiple logistic regression analysis. Subgroup analysis revealed that tirofiban use was significantly associated with favorable outcomes in ACS (aOR = 3.66, 95% CI 1.24–5.22, *p* = 0.019) but not in PCS (aOR = 1.12, 95% CI 0.47–7.52, *p* = 0.570).

**Conclusion:** We demonstrated that tirofiban may be associated with improving favorable outcome for the AIS patients who underwent MT, without increasing ICH or sICH. Furthermore, our results indicated that for PCS patients tirofiban may not be associated with favorable outcome, and more comprehensive randomized controlled trials are needed to confirm this finding.

## Introduction

A number of randomized clinical trials have shown the benefit of mechanical thrombectomy (MT) in the treatment of acute ischemic stroke (AIS) that is due to large-vessel occlusion (LVO) (1, 2). However, this endovascular recanalization approach may lead to endothelial injury, plaque rupture, and subsequent platelet activation, leading to early re-occlusion and poor prognosis. Tirofiban is a non-peptide selective glycoprotein (GP) IIb/IIIa receptor inhibitor that reversibly inhibits fibrinogen-dependent platelet aggregation and subsequent formation of thrombi, which contribute to the major atherosclerotic complications in the progression of AIS (3).

Clinical trials in patients with AIS initially demonstrated the safety and efficacy of tirofiban as an adjunct to MT for AIS patients. However, the results of these trials have been controversial; some studies have shown that tirofiban does not improve prognosis and may even increase intracranial hemorrhage (ICH) and mortality. Therefore, more data are needed to confirm further the benefits and risks of tirofiban. A series of follow-up studies have reported a number of clinically valuable findings about the tirofiban regimen following MT, including the specific dose (4), injection method (5), and patient selection according to etiology (6). However, the specific indications and patient selection are still under debate. Considering the different clinical characteristics of posterior circulation stroke (PCS) and anterior circulation stroke (ACS) (7), as well as the different postoperative prognoses of patients with MT between the two groups (8), there is a hypothesis that tirofiban may have inconsistent risks and benefits in PCS and ACS. At present, there are some studies about the benefit of MT in PCS patients and the administration of tirofiban following MT (9, 10), but there are few studies exploring tirofiban in patients with PCS.

The main aim of this study was to test the safety and efficacy of tirofiban following MT in AIS patients. Propensity score matching (PSM) was used to match tirofiban- and no-tirofiban-treated patients for potential confounders. Regression analysis after PSM was performed to identify independent associations with the outcomes. The secondary aims were to compare the risks and benefits of tirofiban following MT between PCS and ACS.

## Methods

### Patient Selection

In this retrospective study, a total of 292 patients who underwent MT secondary to large artery occlusion (LAO) between January 2015 and May 2019 were enrolled. The inclusion criteria were as follows: (1) patients with AIS secondary to LAO confirmed by computed tomographic angiography (CTA), (2) patients with neurological deficits with a National Institutes of Health Stroke Scale (NIHSS) score of 6 or higher at presentation, (3) patients who were 18 years of age or older, and (4) patients who underwent MT.

The exclusion criteria were as follows: (1) cerebral hemorrhage confirmed by computed tomography; (2) platelet count <100 × 109/L, blood glucose concentration <2.8 or >22.0 mmol/L, and severe hepatic or renal dysfunction; and (3) incomplete data or loss to follow-up. This study was approved by the Ethics Committee of Zhejiang University School of Medicine Second Affiliated Hospital.

### Procedures

All enrolled patients were treated according to current guidelines for AIS and underwent MT employed second-generation stent-retriever devices (Solitaire AB/FR, Covidien/ev3, Irvine, CA; Trevo Proview, Stryker, CA). Alternative rescue therapies, including balloon angioplasty and rescuing stent, were determined by the operators during the procedure based on the characteristics of the lesion and access. Following thrombectomy, all patients underwent postprocedural computed tomography (CT) within 12–24 h, and the presence of hemorrhage on CT was determined and scored by a blinded neuroradiologist.

Tirofiban was considered for application in the following situations: (1) rescue treatment with emergency stenting and balloon angioplasty for residual artery stenosis or failed thrombectomy, (2) successful mechanical recanalization with ≥3 passes with stent retriever, (3) severe *in situ* atherosclerosis with high risk of early re-occlusion, and (4) other recanalization refractory conditions and presumed endothelial damage. Tirofiban was continuously given at a rate of 8 μg/kg·h after an intravenous bolus of 10 μg/kg if there was no evidence of ICH on immediate head CT after MT. Twenty-four hours later, dual antiplatelet therapy was given after ICH was ruled out by another head CT.

### Baseline Assessment and Outcome Measures

Data were extracted through a retrospective review of patient charts, procedure notes, image data, and follow-up notes. Baseline characteristics were collected, including age, sex, presenting NIHSS score, Alberta Stroke Program Early Computed Tomography Score (ASPECTS) or posterior circulation ASPECTS (pc-ASPECTS), comorbidities (diabetes, hypertension, atrial fibrillation, hyperlipidemia, and a history of prior stroke), antiplatelet drug and anticoagulation drug use, and coagulation function indicators. Procedural variables included time from symptom onset to groin puncture, time from symptom onset to reperfusion, tissue plasminogen activator (t-PA) use, retrieval times ≥3, rescue therapy including balloon angioplasty and rescuing stent, and Thrombolysis in Cerebral Infarction (TICI) grading. A TICI grade better than 2b was defined as successful recanalization. The arterial occlusion site was recorded as ACS and PCS. The stroke etiology was classified according to the Trial of ORG 10172 in Acute Stroke Treatment (TOAST) criteria: large artery atherosclerosis (LAA), cardioembolism (CE), and stroke of other determined or undetermined causes.

The primary outcome measure was functional outcome, which was measured by the modified Rankin Scale (mRS) at 3 months. A favorable outcome was defined as an mRS score of 0–2. The scores were collected by a stroke neurologist during routine follow-up visits at 90 days (±14) after stroke for the majority of patients. Telephone discussions with patients or their families were used to obtain information. ICH was considered present when head CT revealed a region consistent with newly developed blood extravasation. Correspondingly, symptomatic intracranial hemorrhage (sICH) was defined as any hemorrhage with neurological deterioration, indicated by an NIHSS score of ≥4 points above the baseline value, or as any hemorrhage leading to mortality. Two investigators, who were blinded to all clinical information, independently reviewed the CT and magnetic resonance imaging (MRI) images to determine the presence of ICH or sICH.

### Statistical Analysis

Statistical analyses were performed using SPSS V.25 for the majority of the data. Patient variables were analyzed using descriptive statistics and univariate comparisons. Comparisons were performed using the *t*-test for continuous measures, for non-continuous variables, and χ^2^ test for categorical measures. All tests were two sided, and an α < 0.05 was considered significant.

PSM was performed with R 4.0.3 (R Foundation for Statistical Computing, Vienna, Austria) to ensure an even distribution of possible confounders between the two groups. A 1:1 matched analysis using nearest-neighbor matching with a caliper distance of 0.1 without replacement was performed based on the estimated propensity score of each patient. After matching patient characteristics, these were analyzed again to confirm successful matching. Multivariate logistic regression analysis was used to assess the odds ratio (OR) and corresponding 95% confidence interval (CI) to explore whether tirofiban can independently affect favorable clinical outcomes (mRS scores 0–2) and safety outcomes, including ICH, sICH, and mortality at 3 months.

## Results

### Patient Characteristics

A total of 292 patients were eligible for this study. The baseline characteristics and outcomes of the patients are presented in Table 1 and were compared between the tirofiban group (*n* = 51) and the no-tirofiban group (*n* = 241). There was no significant between-group difference with respect to age, sex, or NIHSS score at presentation (*p* > 0.05). Coagulation function was assessed by preoperative and postoperative coagulation indicators, including prothrombin time (PT), activated partial thromboplastin time (APTT), and platelet count, which were similar between the two groups. PCS was more common in the tirofiban group (25.5 vs. 12.9%, *p* = 0.038). LAA was the cause of stroke in 74.5% of tirofiban patients, compared with 50.6% in the no-tirofiban group (*p* < 0.001). In contrast, CE was lower in the tirofiban group (29.4 vs. 58.9%, *p* < 0.01). Other medical histories and comorbidities showed no between-group differences.

**Table 1:** Patient characteristics and outcomes before and after PSM.

	**Before PSM**			**After PSM**		
	**Tirofiban**	**No tirofiban**	***p***	**Tirofiban**	**No tirofiban**	***p***
	***n* = 51**	***n* = 241**		***n* = 51**	***n* = 51**	
Age (year)	66.2 ± 11.4	68.5 ± 14.3	0.262	66.2 ± 11.4	66.7 ± 8.7	0.954
Female, *n* (%)	18 (35.3)	97 (40.2)	0.617	18 (35.3)	21 (41.2)	0.541
Involved vessel, *n* (%)			0.038^*^			0.818
ACS	38 (74.5)	210 (87.1)		38 (74.5)	39 (80.4)	
PCS	13 (25.5)	31 (12.9)		13 (25.5)	12 (19.6)	
TOAST, *n* (%)			0.001^*^			0.089
LAA	38 (74.5)	122 (50.6)		38 (74.5)	31 (60.8)	
CE	7 (13.7)	106 (44.0)		7 (13.7)	14 (27.5)	
Other	6 (11.8)	13 (5.4)		6 (11.8)	6 (11.7)	
NIHSS, median (IQR)	14 (11–18)	12 (9–16)	0.078	14 (11–18)	13 (9–18)	0.638
ASPECTS/pc-ASPECTS, median (IQR)	8 (7–9)	8 (7–9)	0.788	8 (7–9)	8 (7–9)	0.832
Systolic BP (mm Hg)	144.6 ± 22.6	144.1 ± 18.1	0.869	144.6 ± 22.6	147.2 ± 18.3	0.553
Glucose (mmol/L)	7.17 ± 2.08	8.69 ± 22.91	0.638	7.17 ± 2.08	7.32 ± 1.84	0.709
**Medical history**
Atrial fibrillation, *n* (%)	15 (29.4)	142 (58.9)	0.001^*^	15 (29.4)	36 (70.6)	0.043^*^
Hyperlipidemia, *n* (%)	1 (2.0)	4 (1.7)	1.000	1 (2.0)	1 (2.0)	1.000
Hypertension, *n* (%)	37 (72.5)	157 (65.1)	0.393	37 (72.5)	35 (68.6)	0.828
Diabetes mellitus, *n* (%)	7 (13.7)	38 (15.8)	0.878	7 (13.7)	9 (17.6)	0.785
Previous stroke, *n* (%)	11 (21.6)	40 (16.6)	0.518	11 (21.6)	8 (15.7)	0.445
Pre-antiplatelet, *n* (%)	6 (11.8)	35 (14.5)	0.769	6 (11.8)	8 (15.7)	0.774
Pre-anticoagulation, *n* (%)	2 (3.9)	21 (8.7)	0.385	2 (3.9)	2 (3.9)	1.000
Smoker, *n* (%)	9 (17.6)	30 (12.4)	0.444	9 (17.6)	4 (7.8)	0.138
**Coagulation function**
Pre-platelet (10^9^/L)	178.8 ± 49.7	178.2 ± 60.5	0.945	178.8 ± 49.7	171.4 ± 53.5	0.476
Post-platelet (10^9^/L)	186.7 ± 61.8	185.7 ± 65.3	0.916	186.7 ± 61.8	183.1 ± 62.8	0.771
**Procedural variables**
t-PA treated, *n* (%)	42 (82.4)	215 (89.2)	0.257	42 (82.4)	46 (90.2)	0.388
Time 1 (min)	414.2 ± 394.3	314.5 ± 172.2	0.083	414.2 ± 394.3	390.8 ± 233.2	0.716
Time 2 (min)	515.7 ± 394.3	393.9 ± 182.9	0.035^*^	515.7 ± 394.3	493.8 ± 241.5	0.738
Retrieval times ≥3, *n* (%)	5 (9.8)	9 (3.7)	0.018^*^	5 (9.8)	1 (2.0)	0.092
Balloon angioplasty, *n* (%)	7 (13.7)	18 (7.5)	0.062	7 (13.7)	2 (3.9)	0.081
Permanent stenting, *n* (%)	6 (11.8)	11 (4.5)	0.046^*^	6 (11.8)	2 (3.9)	0.141
TICI 2b−3, *n* (%)	44 (86.3)	221 (91.7)	0.342	44 (86.3)	43 (84.3)	0.780
**Clinical outcome**
Favorable outcome, *n* (%)	25 (49.0)	87 (36.1)	0.085	25 (49.0)	13(25.49)	0.014^*^
sICH, *n* (%)	9 (17.6)	52 (21.5)	0.531	9 (17.6)	14(27.45)	0.236
ICH, *n* (%)	16 (31.3)	99 (41.0)	0.197	16 (31.3)	23 (45.1)	0.154
Mortality at 3 months, *n* (%)	8 (15.6)	46 (19.0)	0.570	8 (15.6)	17 (33.3)	0.064

*Data are mean ± SD, n (%), or median (IQR). Factors matched by PSM included age, NIHSS score, time from symptom onset to reperfusion, involved vessel site, TOAST classification, retrieval times ≥3, permanent stenting, and ASPECTS.*

* *Statistically significant.*

*PSM, propensity score matching; ACS, anterior circulation stroke; PCS, posterior circulation stroke; TOAST, Trial of ORG 10172 in Acute Stroke Treatment; LAA, large artery atherosclerosis; CE, cardioembolism; NIHSS, National Institutes of Health Stroke Scale; IQR, interquartile range; ASPECTS, Alberta Stroke Program Early Computed Tomography Score; pc-ASPECTS, posterior circulation ASPECTS; BP, blood pressure; t-PA, tissue plasminogen activator; Time 1, time from symptom to groin puncture; Time 2, time from symptom to reperfusion; TICI, Thrombolysis in Cerebral Infarction grading; sICH, symptomatic intracranial hemorrhage; ICH, intracranial hemorrhage*.

The analysis of procedural variables showed that ~88.1% of patients received t-PA, without a significant difference between the two groups. The mean times from symptom onset to recanalization were comparable (414.2 ± 394.3 vs. 314.6 ± 172.2, *p* > 0.05), but the time from symptom onset to reperfusion in the tirofiban group was longer (515.78 ± 394.34 vs. 393.97 ± 182.95, *p* = 0.035). Furthermore, the patients with tirofiban were more often to accept the rescue therapies including balloon angioplasty (13.7 vs. 7.5%, *p* = 0.062) and permanent stenting (11.8 vs. 4.5%, *p* = 0.046) and undergo MT with retrieval times ≥3 (9.8 vs. 3.7%, *p* = 0.018). The overall rate of recanalization was 90.8%, and it was not significantly different between the two groups.

### Safety and Efficacy Outcomes

The efficacy and safety outcome measures were not significantly different. The rates of favorable outcomes (mRS 0–2) were 49.0 and 36.1%, respectively, in the tirofiban and no-tirofiban groups, but *p* > 0.05. The overall mortality at 3 months was 18.50% across both groups and was slightly, but not significantly, lower in the tirofiban group than in the no-tirofiban group (15.6 vs. 19.1%, *p* > 0.05). Procedure-related complications in the tirofiban group, including ICH (31.4 vs. 41.1%, *p* = 0.197) and sICH (17.65 vs. 21.58%, *p* = 0.531), did not occur more frequently than in the no-tirofiban group.

**Figure 1:**
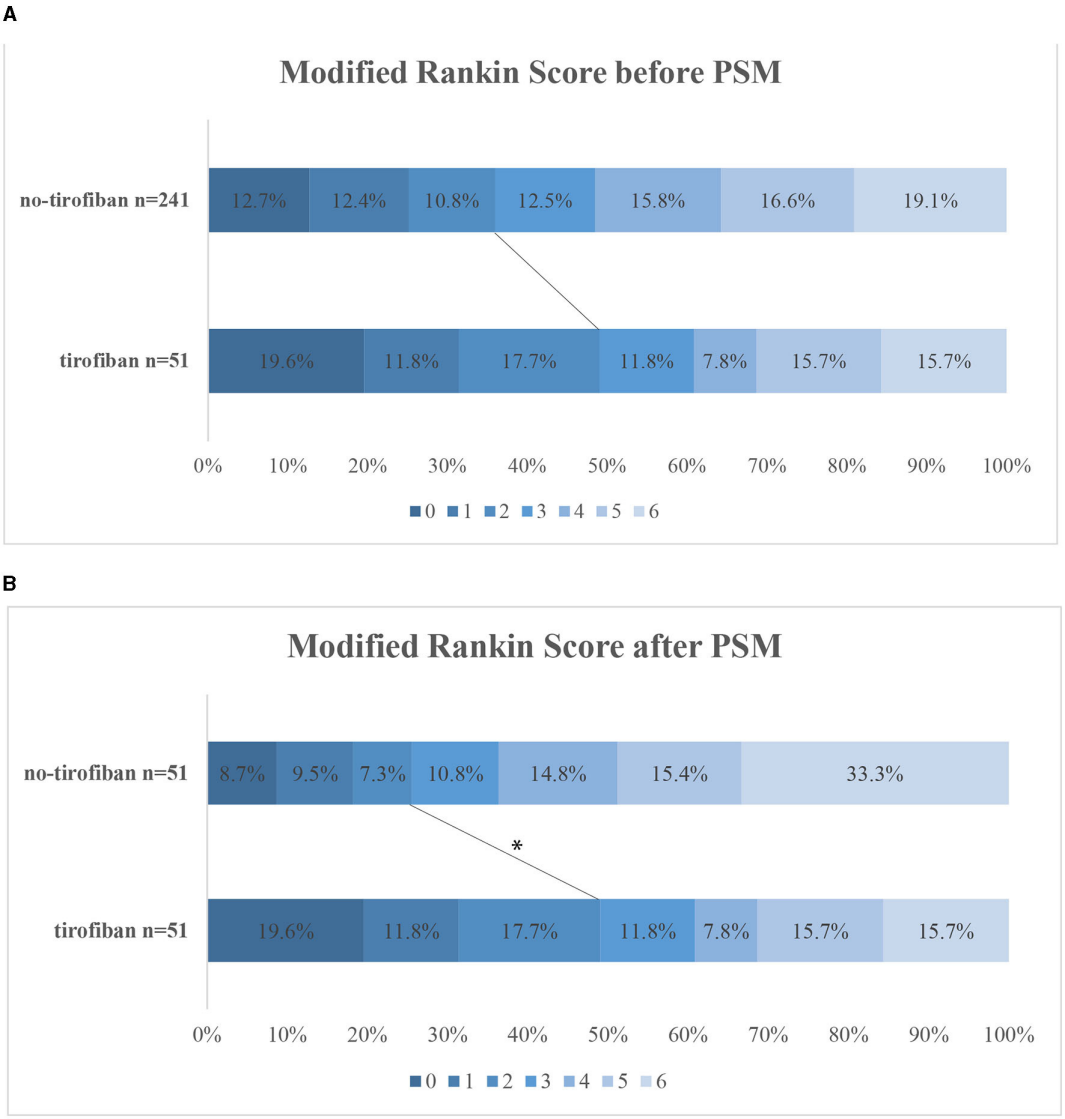
Distribution of mRS at 3 months categories before PSM **(A)** and after PSM **(B)**. The lines indicate differences in favorable outcome (mRS 0–2) between groups. *p < 0.05. mRS, modified Rankin Scale; PSM, propensity score matching.

Variables in the PSM were selected based on previous univariate analysis, including age, NIHSS score, time from symptom onset to reperfusion, involved vessel site TOAST classification, and ASPECTS. Finally, 51 cases were successfully matched, and the standard deviation indicated that the matching effect was satisfactory. After PSM, the characteristics of the two groups were relatively the same after matching (Table 1). After PSM, tirofiban significantly improved the rates of favorable outcomes in the tirofiban and no-tirofiban groups (49.0 vs. 25.5%, *p* = 0.014) (Figure 1). No difference was found in mortality and the rates of ICH and sICH (*p* > 0.05).

Stepwise regression was performed to identify the factors associated with the safety and efficacy outcomes (Supplementary Table 1). In the overall patients, tirofiban tended to improve the rates of favorable outcomes independently [adjusted odds ratio (aOR) = 1.45, 95% CI 1.67–3.43, *p* = 0.024]. Furthermore, tirofiban did not show any association with the incidences of ICH, sICH, or mortality (*p* = 0.605, 0.353, and 0.362, respectively).

The results remained stable after PSM for potential confounders. The use of tirofiban showed an independent association with better outcomes (aOR = 2.87, 95% CI 1.52–6.44, *p* = 0.043). Tirofiban was not associated with a risk of ICH (aOR = 0.67, 95% CI 0.37–2.13, *p* = 0.451), sICH (aOR = 0.55, 95% CI 0.67–1.23, *p* = 0.263), or mortality at 3 months (aOR = 0.45, 95% CI 0.57–1.43, *p* = 0.728). And the multivariate regression models were adjusted, respectively, for the favorable outcome and safety outcome (Table 2).

**Table 2:** Multivariate regression analysis of effects of tirofiban on safety and efficacy outcomes.

	**Before PSM (** ***n*** **=** **294)**		**After PSM (** ***n*** **=** **102)**	
	**aOR (95% CI)**	***p***	**aOR (95% CI)**	***p***
Favorable outcome^a^	1.45 (1.67–3.43)	0.024^*^	2.87 (1.52–6.44)	0.043^*^
ICH^b^	0.24 (0.29–1.19)	0.605	0.67 (0.37–2.13)	0.451
sICH^b^	0.67 (0.27–1.49)	0.353	0.55 (0.67–1.23)	0.263
Mortality at 3 months^b^	0.66 (0.25–1.55)	0.362	0.45 (0.57–1.43)	0.728

a *Model 1 adjusted for age, ASPECTS/pc-ASPECTS, tirofiban, baseline NIHSS, glucose, TOAST classification, and location (posterior or anterior circulation).*

b *Model 2 adjusted for age, ASPECTS/pc-ASPECTS, tirofiban, baseline NIHSS, TOAST classification, involved vessel site (posterior or anterior circulation), and previous stroke.*

* *Statistically significant.*

*PSM, propensity score matching; aOR, adjusted odds ratio; CI, confidence interval; ICH, intracranial hemorrhage; sICH, symptomatic intracranial hemorrhage; ASPECTS, Alberta Stroke Program Early Computed Tomography Score; pc-ASPECTS, posterior circulation ASPECTS; NIHSS, National Institutes of Health Stroke Scale; TOAST, Trial of ORG 10172 in Acute Stroke Treatment*.

### Effects on ACS and PCS

Based on the above regression analysis results, we performed a subgroup analysis according to location to explore further the association between tirofiban and favorable outcomes. First, the main characteristics and clinical outcomes of patients with ACS and PCS were compared with univariate analysis, and there were many differences between the two groups (Table 3). Patients with PCS had higher NIHSS scores on admission than those with ACS (21.9 ± 13.1 vs. 14.4 ± 5.9, *p* = 0.001), a longer time from symptom onset to groin puncture (494.0 ± 448.6 vs. 303.2 ± 146.5, *p* = 0.008), and a longer time from symptom onset to reperfusion (587.0 ± 450.7 vs. 384.7 ± 158.0, *p* = 0.005). A total of 63.6% of patients with PCS had LAA stroke, compared with 52.4% of patients with ACS (*p* = 0.005). Relatedly, tirofiban administration was more common in PCS patients (29.5 vs. 15.3%, *p* = 0.001). In terms of comparison with the outcomes of the two groups, patients in the PCS group had significantly worse favorable clinical outcomes (20.4 vs. 41.5%, *p* = 0.008). There were no significant differences in ICH, sICH, or risk of death between the two groups.

**Table 3:** Patient characteristics and outcomes on patients with ACS and PCS.

	**ACS *n* = 248**	**PCS *n* = 44**	***p***
**Characteristics**			
Age (year)	69 ± 9.8	65 ± 11.3	0.122
NIHSS, *n* (%)	14.4 ± 5.9	21.9 ± 13.1	0.001^*^
ASPECTS/pc-ASPECTS, median (IQR)	8 (7–9)	8 (7–9)	0.436
TOAST, *n* (%)			0.005^*^
LAA	130 (52.4)	28 (63.6)	
CE	104 (42.7)	9 (20.4)	
Other	12 (4.8)	7 (15.9)	
Tirofiban, *n* (%)	38 (15.3)	13 (29.5)	0.022^*^
Time 1 (min)	303.2 ± 146.5	494.0 ± 448.6	0.008^*^
Time 2 (min)	384.7 ± 158.0	587.0 ± 450.7	0.005^*^
TICI 2b−3	229(92.3)	36(81.8)	0.026^*^
**Clinical outcome**			
Favorable outcome, *n* (%)	103 (41.5)	9 (20.4)	0.008^*^
ICH, *n* (%)	100 (40.2)	15 (34.0)	0.436
sICH, *n* (%)	51 (20.5)	10 (22.7)	0.745
Mortality#, *n* (%)	42 (16.9)	12 (27.2)	0.104

*Values are n (%), mean ± SD.*

* *Statistically significant.*

*ACS, anterior circulation stroke; PCS, posterior circulation stroke; NIHSS, National Institutes of Health Stroke Scale; ASPECTS, Alberta Stroke Program Early Computed Tomography Score; pc-ASPECTS, posterior circulation ASPECTS; IQR, interquartile range; TOAST, Trial of ORG 10172 in Acute Stroke Treatment; LAA, large artery atherosclerosis; CE, cardioembolism; Time 1, time from symptom to groin puncture; Time 2, time from symptom to reperfusion; TICI, Thrombolysis in Cerebral Ischemia; ICH, intracranial hemorrhage; sICH, symptomatic intracranial hemorrhage*.

**Figure 2:**
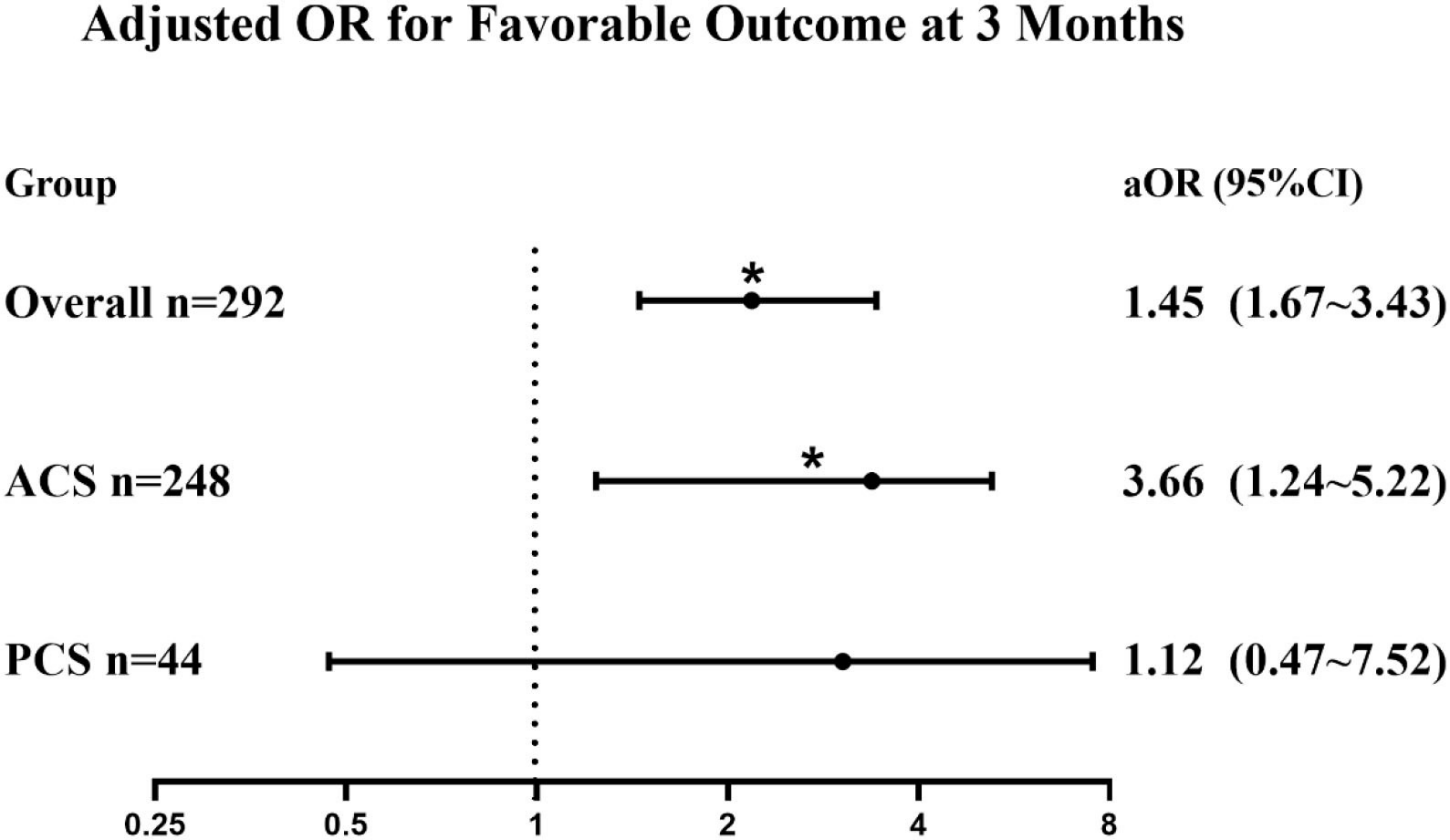
Forest plot of adjusted OR for the association between tirofiban administration and favorable outcomes (mRS 0–2) in patients with posterior circulation stroke (PCS) and anterior circulation stroke (ACS). Adjusted for age, ASPECTS/pc-ASPECTS, tirofiban, baseline NIHSS, glucose, TOAST classification, and location (posterior or anterior circulation). aOR, adjusted odds ratio; mRS, modified Rankin Scale; ASPECTS, Alberta Stroke Program Early Computed Tomography Score; pc-ASPECTS, posterior circulation ASPECTS; NIHSS, National Institutes of Health Stroke Scale; TOAST, Trial of ORG 10172 in Acute Stroke Treatment. *p < 0.05.

A multivariate analysis was performed to adjust the confounders and to identify the use of tirofiban as the independent predictor of favorable outcomes in the overall group (aOR = 1.45, 95% CI 1.67–3.43, *p* = 0.024). In the ACS patients, tirofiban was associated with favorable outcomes (aOR = 3.66, 95% CI 1.24–5.22, *p* = 0.019); however, such an association was not observed in PCS patients (aOR = 1.12, 95% CI 0.47–7.52, *p* = 0.570). Detailed information on the regression coefficients and *p*-values is presented in Supplementary Table 2. The comparison between subgroups is more intuitively reflected in Figure 2.

## Discussion

In this study, we evaluated the safety and efficacy of tirofiban as an adjuvant therapy for MT in AIS patients. The main findings of our study are as follows: (1) more patients in the tirofiban group had favorable clinical outcomes after PSM matching, and ICH and mortality did not differ between the two groups; (2) multivariate regression analysis demonstrated that tirofiban was positively associated with favorable clinical outcomes but not with ICH, mortality, or other safety indicators; and (3) tirofiban was associated with increasing favorable clinical outcomes in patients with ACS but not in patients with PCS.

The purpose of therapy for AIS patients is to achieve rapid cerebral vascular recanalization to restore cerebral blood flow (10), and MT has become the first-line treatment for LVO (11). However, many patients who achieve rapid vascular recanalization by MT are still at high risk of acute reocclusion, especially LAA patients (12, 13). The potential cause may be *in situ* atherosclerotic occlusion, local platelet aggregation, and broken plaques (14). LAA strokes are the most common stroke type in China, so it is urgent to find ways to prevent reocclusion (15).

Tirofiban is a relatively short-acting and reversible GP IIb/IIIa receptor inhibitor that inhibits fibrinogen binding to platelets, effectively preventing platelet aggregation and secondary thrombosis (16). There have been a number of clinical trials demonstrating the safety and efficacy of tirofiban administration in AIS patients undergoing MT, but with controversial results. Most studies have demonstrated that tirofiban has great clinical application prospects in MT, and tirofiban has proved to be an independent predictor of favorable outcomes (17–19). A multicenter retrospective cohort confirmed that the safety of tirofiban was not associated with ICH or mortality (20). In contrast, other studies have shown that tirofiban does not improve clinical outcomes (21) and may be associated with an increased risk of fatal ICH (22, 23). The reasons for the controversial results are complex, including the heterogeneity of experiments, relatively small sample sizes, patient selection bias, and different tirofiban application regimens (5, 24). In our study, tirofiban was continuously given at a rate of 8 μg/kg·h after an intravenous bolus of 10 μg/kg if there was no evidence of ICH. The dose of tirofiban was not consistent in previous studies, but it did not vary extensively. And the total amount of tirofiban infusion was low and only varied from 0.5 to 2.0 mg in most centers (3). Nevertheless, the specific dose may affect the results especially for the risk of ICH, and further research about the dose is needed.

In this study, the use of tirofiban did not lead to significantly more benefits before PSM. We found the patients in the tirofiban group were more often with rescuer therapies, repeated attempts of thrombectomy, and longer time from symptom onset to reperfusion, which were associated with difficulty of vascular recanalization. Additionally, in our study tirofiban was more selectively adopted for patients with LAA stroke rather than CE stroke (74.5 vs. 13.7%, *p* = 0.01). This patient selection bias may explain the lower rate of recanalization in the tirofiban group (86.3 vs. 91.7%, *p* = 0.342). After the correction of these confounding factors by PSM, the rate of recanalization has become similar in the matched cohort, and the use of tirofiban significantly improved the rate of favorable outcomes in AIS patients.

A number of studies have noted the differences between ACS and PCS in clinical characteristics, treatment, and prognosis (25). PCS accounts for 5–19% of AIS patients (26, 27). A large clinical trial including 90,484 patients treated with t-PA demonstrated that PCS was associated with worse outcomes (28). Some studies have suggested that MT for PCS is a safe and feasible treatment option (29). MT is widely used in PCS clinically, and our study results showed that more PCS patients than ACS patients were treated with tirofiban (29.5 vs. 15.3%). However, few studies have focused on whether tirofiban administration in MT has consistent safety and efficacy between ACS and PCS. Subgroup analysis was performed, and we found that PCS patients had an obviously worse prognosis than ACS patients (20.4 vs. 41.5%, respectively), and they also had a higher NIHSS score at admission and a longer time from symptom onset to reperfusion. Further analysis by logistic regression illustrated that tirofiban was associated with increasing favorable clinical outcomes in patients with ACS but not in patients with PCS. However, Alawieh et al. thought that patients with PCS benefit equally from tirofiban administration compared with ACS (30). Given the limited sample size, our results only indicated that the effect of tirofiban may be modified by the occlusion sites and the PCS patients seemed to benefit less. Whether tirofiban treatment can affect efficacy outcomes of PCS patients treated with MT requires future research.

There are also the limitations in this study. First, this experiment included only patients in the past 5 years from a single center, which reduced the changes and differences in perioperative patient care procedures but also resulted in a small sample size. Additionally, this is a retrospective study with inevitable patient selection bias. Even though we used advanced statistical methods, including PSM and multivariate adjustment, we cannot correct residual or unmeasured confounding. Furthermore, the balloon angioplasty and stenting are more commonly used in tirofiban groups, which may exaggerate the effect of tirofiban. Considering these limitations, these results should be analyzed with more caution, and larger multicenter data will be required to study this effect further.

## Conclusion

We demonstrated that tirofiban may be associated with improving favorable outcome for the AIS patients who underwent MT, without increasing ICH or sICH. Furthermore, our results indicated that for PCS patients tirofiban may not be associated with favorable outcome, and more comprehensive randomized controlled trials are needed to confirm this finding.

## Data Availability Statement

The raw data supporting the conclusions of this article will be made available by the authors, without undue reservation.

## Author Contributions

LC, LX, and JX collected the data and drafted the manuscript. JY, CL, and XY analyzed the data and performed all statistical analyses. ML, CY, and CQ conceived the study and made critical revisions to the manuscript. All authors contributed to the article and approved the submitted version.

## Funding

This work was supported by the National Natural Science Foundation of China (Grant nos. 81701152 and 81971097), Natural Science Foundation of Zhejiang Province (LY19H090022), and Medical and Health Science and Technology Program of Zhejiang Province (2016KYA199).

## Conflict of Interest

The authors declare that the research was conducted in the absence of any commercial or financial relationships that could be construed as a potential conflict of interest.

## Publisher's Note

All claims expressed in this article are solely those of the authors and do not necessarily represent those of their affiliated organizations, or those of the publisher, the editors and the reviewers. Any product that may be evaluated in this article, or claim that may be made by its manufacturer, is not guaranteed or endorsed by the publisher.

## References

[1] Martins SO Mont'Alverne F Rebello LC Abud DG Silva GS Lima FO. Thrombectomy for stroke in the public health care system of Brazil. N Engl J Med. (2020) 382:2316–26. 10.1056/NEJMoa2000120 32521133

[2] Saver J Goyal M Bonafe A Diener H Levy E Pereira V. Stent-retriever thrombectomy after intravenous t-PA vs. t-PA alone in stroke. N Engl J Med. (2015) 372:2285–95. 10.1056/NEJMoa1415061 25882376

[3] Yang M Huo X Miao Z Wang Y. Platelet Glycoprotein IIb/IIIa receptor inhibitor tirofiban in acute ischemic stroke. Drugs. (2019) 79:515–29. 10.1007/s40265-019-01078-0 30838514

[4] Zhao W Che R Shang S Wu C Li C Wu L. Low-dose tirofiban improves functional outcome in acute ischemic stroke patients treated with endovascular thrombectomy. Stroke. (2017) 48:3289–94. 10.1161/STROKEAHA.117.019193 29127270

[5] Yang J Wu Y Gao X Bivard A Levi C Parsons M. Intraarterial versus intravenous tirofiban as an adjunct to endovascular thrombectomy for acute ischemic stroke. Stroke. (2020) 51:2925–33. 10.1161/STROKEAHA.120.029994 32933416

[6] Sun B Shi Z Pu J Yang S Wang H Yang D. Effects of mechanical thrombectomy for acute stroke patients with etiology of large artery atherosclerosis. J Neurol Sci. (2019) 396:178–83. 10.1016/j.jns.2018.10.017 30476651

[7] Kim JT Park MS Choi KH Kim BJ Han MK Park TH. Clinical outcomes of posterior versus anterior circulation infarction with low national institutes of health stroke scale scores. Stroke. (2017) 48:55–62. 10.1161/STROKEAHA.116.013432 27856952

[8] Caplan L Wityk R Glass T Tapia J Pazdera L Chang H. New England medical center posterior circulation registry. Ann Neurol. (2004) 56:389–98. 10.1002/ana.20204 15349866

[9] Weber R Minnerup J Nordmeyer H Eyding J Krogias C Hadisurya J. Thrombectomy in posterior circulation stroke: differences in procedures and outcome compared to anterior circulation stroke in the prospective multicentre REVASK registry. Eur J Neurol. (2019) 26:299–305. 10.1111/ene.13809 30218610

[10] Berkhemer O Fransen P Beumer D van den Berg L Lingsma H Yoo A. A randomized trial of intraarterial treatment for acute ischemic stroke. N Engl J Med. (2015) 372:11–20. 10.1056/NEJMoa1411587 25517348

[11] Yajima H Kanaya H Ogino M Ueki K Kim P. Middle meningeal artery embolization for chronic subdural hematoma with high risk of recurrence: a single institution experience. Clin Neurol Neurosurg. (2020) 197:106097. 10.1016/j.clineuro.2020.106097 32841822

[12] Kang D Yoon W Kim S Baek B Lee Y Kim Y. Endovascular treatment for emergent large vessel occlusion due to severe intracranial atherosclerotic stenosis. J neurosurg. (2018) 130:1−8. 10.3171/2018.1.JNS172350 29932374

[13] Kim Y Sohn S Yoo J Hong J Kim C Kang D. Local tirofiban infusion for remnant stenosis in large vessel occlusion: tirofiban ASSIST study. BMC Neurol. (2020) 20:284. 10.1186/s12883-020-01864-4 32689957 PMC7370431

[14] Mosimann P Kaesmacher J Gautschi D Bellwald S Panos L Piechowiak E. Predictors of unexpected early reocclusion after successful mechanical thrombectomy in acute ischemic stroke patients. Stroke. (2018) 49:2643–51. 10.1161/STROKEAHA.118.021685 30355192

[15] Wang W Jiang B Sun H Ru X Sun D Wang L. Prevalence, incidence, and mortality of stroke in china: results from a nationwide population-based survey of 480 687 adults. Circulation. (2017) 135:759–71. 10.1161/CIRCULATIONAHA.116.025250 28052979

[16] Sun X Zhang H Tong X Gao F Ma G Miao Z. Effects of periprocedural tirofiban vs. oral antiplatelet drug therapy on posterior circulation infarction in patients with acute intracranial atherosclerosis-related vertebrobasilar artery occlusion. Front Neurol. (2020) 11:254. 10.3389/fneur.2020.00254 32351442 PMC7174752

[17] Yang M Huo X Gao F Wang A Ma N Shi H. Low-dose rescue tirofiban in mechanical thrombectomy for acute cerebral large-artery occlusion. Eur J Neurol. (2020) 27:1056–61. 10.1111/ene.14170 32048389

[18] Yi HJ Sung JH Lee DH. Safety and efficacy of intra-arterial tirofiban injection during mechanical thrombectomy for large artery occlusion. Curr Neurovasc Res. (2019) 16:416–24. 10.2174/1567202616666191023154956 31702492

[19] Huo X Yang M Ma N Gao F Mo D Li X. Safety and efficacy of tirofiban during mechanical thrombectomy for stroke patients with preceding intravenous thrombolysis. Clin Interv Aging. (2020) 15:1241–8. 10.2147/CIA.S238769 32801672 PMC7398880

[20] Zhao L Jian Y Li T Wang H Lei Z Sun M. The safety and efficiency of tirofiban in acute ischemic stroke patients treated with mechanical thrombectomy: a multicenter retrospective cohort study. Biochem Res Int. (2020) 2020:5656173. 10.1155/2020/5656173 32399299 PMC7211241

[21] Pan X Zheng D Zheng Y Chan P Lin Y Zou J. Safety and efficacy of tirofiban combined with endovascular treatment in acute ischaemic stroke. Eur J Neurol. (2019) 26:1105–10. 10.1111/ene.13946 30793464

[22] Kellert L Hametner C Rohde S Bendszus M Hacke W Ringleb P. Endovascular stroke therapy: tirofiban is associated with risk of fatal intracerebral hemorrhage and poor outcome. Stroke. (2013) 44:1453–5. 10.1161/STROKEAHA.111.000502 23463755

[23] Wu Y Yin C Yang J Jiang L Parsons M Lin L. Endovascular thrombectomy: tirofiban increases bleeding risk in acute stroke patients. Stroke. (2018) 49:2783–5. 10.1161/STROKEAHA.118.022919 30355186

[24] Goyal M Menon B van Zwam W Dippel D Mitchell P Demchuk A. Endovascular thrombectomy after large-vessel ischaemic stroke: a meta-analysis of individual patient data from five randomised trials. Lancet. (2016) 387:1723–31. 10.1016/S0140-6736(16)00163-X 26898852

[25] Dornák T Král M Sedláčková Z Šanák D Cecháková E Divišová P. Predictors for intracranial hemorrhage following intravenous thrombolysis in posterior circulation stroke. Transl Stroke Res. (2018) 9:582–8. 10.1007/s12975-018-0608-0 29333567

[26] Frid P Drake M Giese AK Wasselius J Schirmer MD Donahue KL. Detailed phenotyping of posterior vs. anterior circulation ischemic stroke: a multi-center MRI study. J Neurol. (2020) 267:649–58. 10.1007/s00415-019-09613-5 31709475 PMC7035231

[27] Sarraj A Medrek S Albright K Martin-Schild S Bibars W Vahidy F. Posterior circulation stroke is associated with prolonged door-to-needle time. Int J Stroke. (2013) 10:672–8. 10.1111/j.1747-4949.2012.00952.x 23521891

[28] Sommer P Posekany A Serles W Marko M Scharer S Fertl E. Is functional outcome different in posterior and anterior circulation stroke? Stroke. (2018) 49:2728–32. 10.1161/STROKEAHA.118.021785 30355215

[29] Meyer L Stracke C Jungi N Wallocha M Broocks G Sporns P. Thrombectomy for primary distal posterior cerebral artery occlusion stroke: the TOPMOST study. JAMA Neurol. (2021) 78:434–44. 10.1001/jamaneurol.2021.0001 33616642 PMC7900924

[30] Alawieh A Vargas J Turner RD Turk AS Chaudry MI Lena J. Equivalent favorable outcomes possible after thrombectomy for posterior circulation large vessel occlusion compared with the anterior circulation: the MUSC experience. J NeuroInterv Surg. (2018) 10:735–40. 10.1136/neurintsurg-2017-013420 29222394

