# Supplementary material for: Can Tirofiban Improve the Outcome of Patients With Acute Ischemic Stroke: A Propensity Score Matching Analysis
Source: Front Neurol. 2021 Sep 13;12:688019. doi: 10.3389/fneur.2021.688019 (PMC8475187; doi:10.3389/fneur.2021.688019)
Supplement: Supplementary file 1 [file Data_Sheet_1.docx]

**Supplement Table 1The predictors of outcomes.**

|  | Favorable outcome | | | ICH | | | sICH | | | Mortality | | |
| --- | --- | --- | --- | --- | --- | --- | --- | --- | --- | --- | --- | --- |
|  | OR | 95% CI | P | OR | 95% CI | P | OR | 95% CI | P | OR | 95% CI | P |
| Age | 0.961 | 0.933 ~ 0.990 | 0.010* | 1.013 | 0.989 ~ 1.038 | 0.292 | 1.024 | 0.990 ~ 1.058 | 0.169 | 1.058 | 1.018 ~ 1.100 | 0.005* |
| ASPECTS# | 1.980 | 1.556 ~ 2.521 | 0.000* | 0.727 | 0.624 ~ 0.847 | 0.000* | 0.669 | 0.561 ~ 0.797 | 0.000* | 0.701 | 0.587 ~ 0.837 | 0.000* |
| TOAST | 0.890 | 0.599 ~ 1.322 | 0.564 | 1.250 | 0.928 ~ 1.684 | 0.143 | 1.140 | 0.798 ~ 1.629 | 0.472 | 1.124 | 0.751 ~ 1.682 | 0.570 |
| Vessel site | 0.624 | 0.213 ~ 1.825 | 0.389 | 0.745 | 0.325 ~ 1.712 | 0.488 | 0.882 | 0.334 ~ 2.332 | 0.801 | 0.748 | 0.272 ~ 2.053 | 0.573 |
| Tirofiban | 2.827 | 1.026 ~ 7.789 | 0.044* | 0.562 | 0.243 ~ 1.299 | 0.178 | 0.700 | 0.253 ~ 1.943 | 0.494 | 0.682 | 0.229 ~ 2.031 | 0.492 |
| NIHSS | 0.915 | 0.862 ~ 0.971 | 0.004* | 1.016 | 0.975 ~ 1.060 | 0.447 | 1.050 | 0.999 ~ 1.105 | 0.057* | 1.070 | 1.014 ~ 1.129 | 0.013* |
| SystolicBP | 0.983 | 0.965 ~ 1.002 | 0.084 | 1.000 | 0.985 ~ 1.016 | 0.955 | 1.013 | 0.994 ~ 1.031 | 0.184 | 1.015 | 0.996 ~ 1.035 | 0.131 |
| Glucose | 0.798 | 0.649 ~ 0.982 | 0.033* | 0.994 | 0.968 ~ 1.021 | 0.658 | 0.999 | 0.976 ~ 1.023 | 0.943 | 0.997 | 0.974 ~ 1.020 | 0.785 |
| Pre-stroke | 1.068 | 0.402 ~ 2.836 | 0.895 | 0.620 | 0.274 ~ 1.402 | 0.251 | 0.338 | 0.108 ~ 1.056 | 0.062* | 0.511 | 0.170 ~ 1.537 | 0.232 |
| Time1 | 1.004 | 0.995 ~ 1.013 | 0.433 | 0.999 | 0.992 ~ 1.007 | 0.869 | 0.997 | 0.988 ~ 1.006 | 0.543 | 1.003 | 0.993 ~ 1.013 | 0.587 |
| Time2 | 0.996 | 0.987 ~ 1.004 | 0.347 | 1.001 | 0.994 ~ 1.008 | 0.815 | 1.003 | 0.994 ~ 1.012 | 0.545 | 0.998 | 0.987 ~ 1.008 | 0.633 |
| TICI2b3 | 2.624 | 0.534 ~ 12.908 | 0.235 | 1.606 | 0.513 ~ 5.034 | 0.416 | 1.605 | 0.418 ~ 6.163 | 0.491 | 0.646 | 0.177 ~ 2.352 | 0.507 |

ASPECTS#: Alberta Stroke Program Early Computed Tomography Score or posterior circulation ASPECTS; BP, blood pressure; Time 1, time from symptom to groin puncture; Time 2, time from symptom to reperfusion; TICI: Thrombolysis in Cerebral Infarction grading;

#Model 1 adjusted for age, ASPECTS/pc-ASPECTS, tirofiban, baseline NIHSS,Glucose, TOAST classification, Location (posterior or anterior

circulation)

†Model 2 adjusted forage, ASPECTS/pc-ASPECTS, tirofiban, baseline NIHSS, TOAST classification, involved vessel site (posterior or anterior

circulation),previous stroke

**Supplement Table 2 Effects of tirofiban treatment on patients with ACS and PCS.**

|  | No.(n) | Tirofiban  n (%) | No tirofiban  n (%) | aOR（95% CI） | P value |
| --- | --- | --- | --- | --- | --- |
| Overall | 292 | 51 | 241 | 1.45 (1.67~3.43) | 0.024* |
| ACS | 248 | 38 (74.5) | 210 (80.4) | 3.66 (1.24~5.22) | 0.019* |
| PCS | 44 | 13 (29.5) | 31 (70.4) | 1.12 (0.47~7.52) | 0.570 |

Values are n (%); * statistically significant; ACS, anterior circulation stroke; PCS, posterior circulation stroke;

Adjusted for age, ASPECTS/pc-ASPECTS, tirofiban, baseline NIHSS, Glucose, TOAST classification, Location (posterior or anterior circulation)
